# A mechanical evaluation of micro-HA/CS composite scaffolds with interconnected spherical macropores

**DOI:** 10.1186/s12938-015-0114-5

**Published:** 2016-02-02

**Authors:** Li Ruixin, Li Dong, Zhao Bin, Li Hao, Leng Xue, Shi Caihong, Su Weihua, Qin Xiaoli, Yuan Yinghai, An Weining, Zhang Xizheng

**Affiliations:** Institute of Medical Equipment, The Academy of Military Medical Sciences, Tianjin, 300161 China; Tianjin Medical University, Qi Xiangtai Road No. 22, Heping District, Tianjin, 300070 China; NO.1 Hospital of Jilin University, Xinmin District, Changchun, Jinlin, 130000 China

**Keywords:** Micro-HA, CS, Paraffin spheres, Cyclic loading

## Abstract

**Background:**

In the process of bone defective reparation and engineered bone tissue construction, osteoblasts are adhered to the surface of the scaffold materials and impart the external mechanical load to the osteoblasts. So, the dynamic mechanical property of the scaffolds play an important role in the bone tissue repair and it is valuable to research. Material type and the architectural design of scaffolds are also important to facilitate cell and tissue growth. The aim of this study was to prepare a kind of material with good pore connectivity and analyze its dynamic mechanical property.

**Methods:**

Fabrication and characterization of micro-hydroxyapatite(m-HA)/chitosan(CS) polymer composite scaffolds with well interconnected spherical pore architectures were reports. Micro-HA was prepared by being calcined and ball milled. Paraffin spheres in the range of 160–330 µm were fabricated with a dispersion method and used as the porogen in the fabrication of the scaffolds. Polymer scaffolds were fabricated by the technique of compression molding and particulate leaching method. The effects of the porogen content on the properties of the scaffolds were studied.

**Results:**

With the increase of porogen, the pore of the scaffolds increased and became interconnected. Cyclic loading of three scaffolds were tested with 10 % strain under four levels of loading frequency, 0.1, 0.5, 1 and 1.5 Hz. The porous composite scaffolds exhibited a viscosity-elastic behaviour with a maximum stress of 3–4 kPa. At each frequency, modulus value is decreased with the paraffin microspheres content, but there was no significance difference in the peak stress of the three samples. All the samples tested displayed clear hysteresis loops. There was no significance difference in the peak hysteresis of the three samples, and the hysteresis difference values between the sixth compression cycle and the initial cycle for three samples was similar, with no statistically significant differences.

**Conclusions:**

Micro-HA/CS composite scaffolds with interconnected spherical macropores were fabricated using pherical paraffin as porogen. The porous composite scaffolds exhibited a viscosity-elastic behaviour with good repeatability. It is benefit to study the influence of the mechanical load on the cell of the scaffold.

## Background

Porous scaffold is a key component of tissue engineering [1], it plays an important role in the formation of new tissues and provides a temporary scaffold to guide new tissue in growth and regeneration [2, 3].

It is well known that hydroxyapatite is the fundamental inorganic component in human hard tissue [4, 5]. It has been used successfully for bone repair and regeneration, owing to its biocompatible, bioactive, osteoconductive, non-toxic, non-inflammatory and non-immunogenic properties [6, 7].

Chitosan, a natural polysaccharide composed of β-(1 → 4)-linked 2-amino-2-deoxy-d-glucopyranose (glucosamine) and 2-acetamido-2-deoxy-d-glucopyranose (acetylglucosamine), can easily be obtained from chitin, an abundant and renewable biopolymer in the biosphere. Because of its biocompatible, biodegradable and bioactive [8], chitosan has attracted considerable attention in pharmaceutical and biomedical applications [9]. The ability of chitosan to support cell attachment and proliferation is attributed to its chemical properties. The polysaccharide backbone of chitosan is structurally similar to glycosaminoglycans, the major component of the extracellular matrix of bone and cartilage [10].

Incorporation of HA with CS could improve the bioactivity and the bone bonding ability of the CS/HA composites [11]. CS just plays a role of adhesive to dissolve the problem of HA shaping and migration of HA powder when implanted.

As well as material type, the architectural design of scaffolds is important to facilitate cell and tissue growth. Presently, several methods have been developed in preparation of hydroxyapatite composite porous scaffolds based on mixing, co-precipitation and coating methods [12–15]. The above approaches led to the incorporation of inorganic fillers into the structure of composites in the form of either nano- or micro-sized particles. Of them, particulate leaching method provides easy control of pore structure and has been widely employed in the fabrication of scaffolds [16–18]. The first report of a well-defined spherical pore structure with excellent interconnectivity was given by Ma et al. [19, 20], in which paraffin spheres were employed to prepare highly porous scaffolds. Then, Zhang reported on the comparison study of the spherical pores resulting from paraffin and the cubic pores resulting from inorganic salts [21]. As the frame of body, bone tissue endlessly accommodates its quality, density and internal structure in whole vital process. Osteoblast is the main mechanical sensitively cell to response stress and strain signal. In the process of bone defective scaffolds reparation and engineered bone tissue construction, osteoblast adherent growth. As the main body to receive the external irritant, scaffold impart the irritant to the osteoblast. So, the dynamic mechanical property of the scaffolds play an important role in the bone tissue repair and it is valuable to research.

In the present study, CS/HA composite scaffolds were fabricated using pherical paraffin as porogen. The dynamic mechanical properties of these scaffolds with different ratio porogen but a similar pore size range were studied in a comparative way together with the scaffold morphologies.

## Methods

### Materials

CS (200–800 cP, 1 % in 1 % acetic acid, Brookfield (lit.), degree of deacetylation is 75–85 %) was purchased from the Sigma Chemical Company and used without further purification. Paraffin with a melting point between 53 and 57 °C, gelatin, and *N*-hexane were obtained from Tianjin Kemiou Chemical Reagent Company.

### Preparation and characterization

#### HA fabrication and characterization

HA can be produced from natural resources like corals, bovine bone, porcine bone [22] or cuttlefish bone. High temperature heat-treatment of animal bones was found to be most often used technique to produce natural HA. The porcine cancellous bone was collected from nearby butcher shops and all of the attached meat and fat were removed and cleaned from the bones. The ends of the cancellous bone were cut into slices about 2 mm, then cyclic soaked in hydrogen peroxide and ethyl ether to be degreased, deproteined, then being washed and dried. The dried degreased and deproteined bone was calcined (1000 °C, 3 h) to prepare the true bone ceramic (TBC), then the TBC was being ball milled at 230 r/min for 2.5 h.

Fourier transform infrared (FTIR) spectroscopy (NICOLET380 FT-IR) was performed in the wave number range 4000–400 cm^−1^. The sample was mixed with KBr and pressed into a pellet. The solid pellet was used for FTIR spectroscopy.

X-ray diffraction (XRD) (D8 DISCOVER, BRUKER company, German) was carried out to determine the crystal phases of the HA using monochromatic Cu Ka radiation at 40 kV. The 2θ scan range was 10–45°.

The particle size of HA was detected by laser particle size analysator. (LSPOP(6), OMEC company, Zhuhai, China).

#### Preparation of paraffin spheres

Paraffin spheres were prepared by emulsifying the paraffin in a heated 0.5 % (g/ml) gelatin solution and quenching the associated emulsion in ice water. 4 g gelatin was dissolved in 800 mL heated deionized water approximately 80 °C, and then 40 g paraffin was added into the solution. The mixture was mechanically stirred at 350 rpm to form a well dispersed suspension. Two hours later, 600 mL ice water was poured into the stirred suspension to solidify paraffin spheres. The suspension containing the paraffin spheres was then separated and subsequently rinsed with deionized water for several times. The paraffin spheres were sieved by standard sieves (50–90 mesh), after being dried in air, the paraffin spheres were collected and stored in a vacuum desiccator for further use.

The paraffin spheres were observed under the optical microscope (Olympus BX51, Japan) and the images were captured by a digital camera (Olympus DP71, Japan).

#### Preparation of micro-HA/CS composite porous scaffolds

**Fig. 1:**
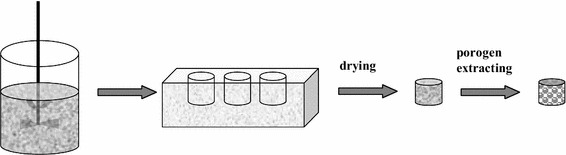
Schematic diagram of micro-HA/CS composite scaffolds shaping method

Paraffin microspheres were used as the porogen in the fabrication of the scaffolds. Polymer scaffolds were fabricated by the technique of compression molding and particulate leaching method [21, 23]. The fabrication scheme of scaffolds was shown in Fig. 1.

In brief, micro-HA was dispersed in deionized water by ultrasonication, then CS and acetic acid was added (The final acetic acid concentration of the solution was 1 %.) under stirring for 1–2 h at room temperature until a homogeneous solution was obtained. Sieved porogen particles were added into the polymer solution to form a paste-like mixture which was then pressed into a PTFE mould and kept in a vacuum oven for 72 h to remove residual solvents. The composite sample was then taken from the mould and the porogen particles were removed using Soxhlet extractor with *N*-hexane as the refluxing solvent for 24 h. The resulting porous scaffolds were air-dried for 24 h, vacuum-dried for another 24 h and stored in a desiccator until further characterization.

### Characterization

#### Scanning electron microscopy (SEM) observation

SEM was carried out to determine the effects of the paraffin spheres content on the pore structure of the composite scaffolds. Slices were cut from the porous scaffolds using a sharp blade for cross-section observation by SEM (LEO 1530VP, German) at an accelerating voltage of 10 kV. The samples were coated with gold prior to SEM observation.

#### Porosity measurement

The porosities of the scaffolds were determined by X-ray microcomputed tomography (Micro-CT) using a SkyScan 1172 system (Skyscan, Belgium) at 7 μm spatial resolution with an integration time of 2 s.

#### Water absorption rate

A known weight of dry scaffold (W_dry_) was immersed in a 6 well cell culture plate filled with 0.1 M PBS solution at room temperature. The samples (n = 6) were removed from the 6 well cell culture plate after 24 h and weighed (W_wet_). Water absorption rate of the scaffolds was calculated by the following equation:

$$ {\text{Water absorption rate}} = \left( {{\text{W}}_{\text{wet}} - {\text{W}}_{\text{dry}} } \right)/{\text{W}}_{\text{dry}} $$

#### Mechanical compress testing: stress–strain cyclic loading

The samples were tested on an Instron Model 5865 Materials Testing Machine (Instron Co., USA) at the ambient temperature. A thickness gauge was used to measure the thickness of each sample in four locations and the average thicknesses were used as inputs in the resulting stress–strain analysis. Cuboid scaffolds of 10 mm in width and 2–5 mm in height were compressed.

For all of the cyclic loading tests conducted, deformations of the samples were defined by the strain ε = ∆L/L_0_, where ∆L is the decrease in sample thickness relative to the initial thickness, L_0_. The resulting stress on each sample is defined by σ = F/A, where F is the compressive force and A is the cross-sectional area of the sample. In order to compare the time-dependent behavior, each cyclic loading test consisted of 15 compression cycles. Applied a preload of 0.1 N and compressive strain of 10 % was chosen for all samples.

Additionally, each sample was tested under four levels of loading frequency, 0.1, 0.5, 1 and 1.5 Hz. For each material group three samples were tested and the average and standard deviation were calculated. Samples were compressed under a sinusoidal strain defined by ε = ∆ε sin(ωt), where ∆ε and ω are the respective strain amplitude and frequency. The response (a strain) though sinusoidal is not in phase with the developed stress, and lags behind the stress by phase angle 90. Reproducible stress–strain curves were plotted and showed in the display. Results of the cyclic loading tests were analyzed by calculating both the instantaneous and steady-state tangent moduli, E_int_ and E_ss_, which correspond to the peak stresses at the first and last cycle relative to the respective strain amplitudes.

### Hysteresis analysis

Hysteresis of the energy dissipating capabilities is a fundamental property of all viscoelastic tissues. In order to determine how the paraffin spheres content affected the mechanical property of composite scaffolds, integrals were carried out over each hysteresis loop and were recorded as the amount of energy dissipation per unit volume of material.

### Statistical analysis

SPSS11.5 was used to evaluate the significant differences among the six groups. Data were presented as mean ± standard error. In all cases, the results were considered statistically different at p < 0.05.

## Results

### HA characterization

**Fig. 2:**
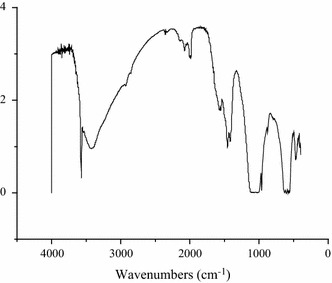
FTIR spectra of HA

The spectra is showed in Fig. 2, where strong absorption bands around 570–600 and 1050 cm^−1^ corresponding to the ${\text{PO}}_{4}^{3 - }$ ions of the apatite. These are assigned to the stretching and bending vibrations of ${\text{PO}}_{4}^{3 - }$group. The absorption band at 600 cm^−1^ results from the ν_4_ mode of O–P–O bending vibration in apatite, whereas the peaks at 1126 and 1020 cm^−1^ indicate the ν_4_ band of P–O stretching mode. The shoulder at 970 cm^−1^ reflects the ν_1_ band of P–O stretching mode in apatite.

**Fig. 3:**
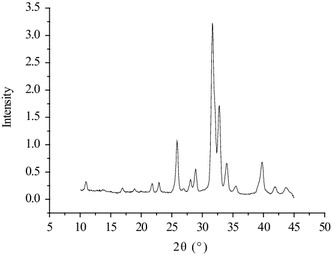
XRD spectra of HA

XRD spectrum of HA is showed in Fig. 3, in which diffraction peak at 25.8, 31.8 and 39.6° corresponding to (002), (211) and (310) of HA.

**Fig. 4:**
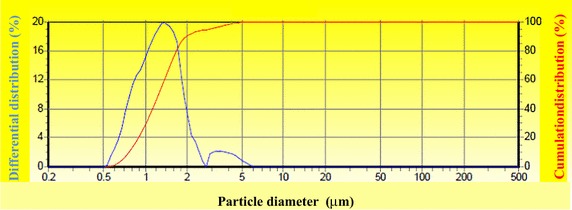
Particle size distribution of micro-HA

Particle size distribution of the HA particles obtained is shown in Fig. 4, and the variations of diameters, differential distribution and cumulation distribution for HA particles are given in Table 1. The treated HA particles mainly distributed in the range of 1 ~ 2 μm and showed a relatively narrow and uniform size distribution.

**Table 1:** HA particle size distribution

Particle size (μm)	Differential distribution (%)	Cumulation distribution (%)	Particle size (μm)	Differential distribution (%)	Cumulation distribution (%)
0.20			2.89	1.63	94.56
0.24	0.00	0.05	3.50	2.14	96.70
0.29	0.00	0.05	4.24	1.87	98.58
0.35	0.00	0.05	5.13	1.03	99.61
0.43	0.00	0.05	6.21	0.38	99.99
0.52	0.00	0.05	7.51	0.01	100.00
0.63	2.84	2.89	9.09	0.00	100.00
0.76	7.70	10.58	11.00	0.00	100.00
0.92	12.17	22.75	13.31	0.00	100.00
1.11	15.46	38.21	16.11	0.00	100.00
1.35	19.84	58.05	19.50	0.00	100.00
1.63	18.55	76.60	23.60	0.00	100.00
1.97	11.21	87.81	28.56	0.00	100.00
2.39	5.13	92.93	34.57	0.00	100.00

### Observation of paraffin spheres

**Fig. 5:**
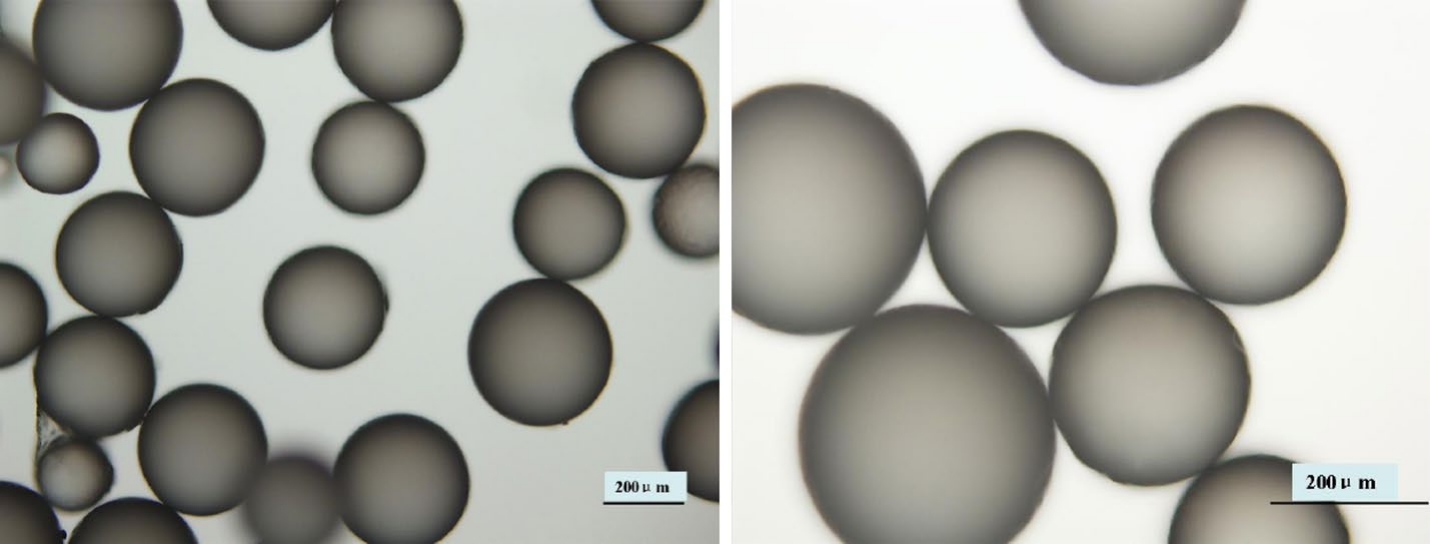
Optical microscopic images of spherical porogens

Mechanical stirring and subsequent quenching of the paraffin suspension with ice water produced uniform spherical paraffin particles (Fig. 5). The size of the most spheres was controlled in the range of 160 ~ 330 *µ*m, and the average size is (292 ± 50) *µ*m.

### SEM observation of scaffolds

**Fig. 6:**
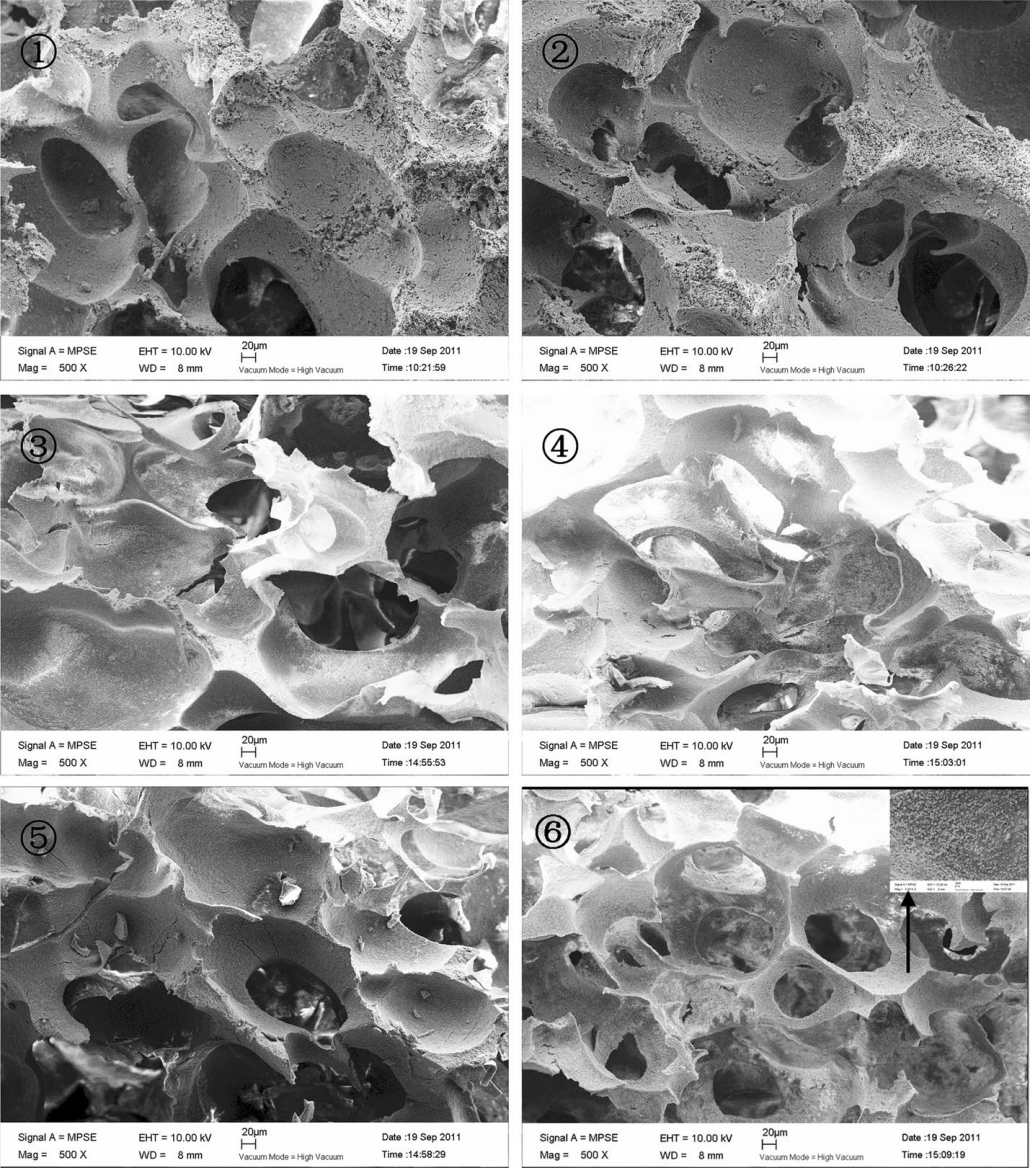
SEM micrographs of composite scaffolds prepared by the particulate leaching method: V_composition_:V_microspheres_ ➀ 50:20 ➁ 50:30 ➂ 50:40 ➃ 50:50 ➄ 50:60 ➅ 50:70

Taking into account the interconnected macroporosity as a requisite for successful tissue engineering scaffolds, a combination compression molding and particulate leaching method is needed. Composition solutions and paraffin spheres of several different volume ratios (50:20, 50:30, 50:40, 50:50, 50:60, 50:70) were tested to find an optimal porogen concentration to obtain interconnected spherical pore network. SEM micrographs of composite scaffolds are showed in Fig. 6.

### Porosity measurement

**Fig. 7:**
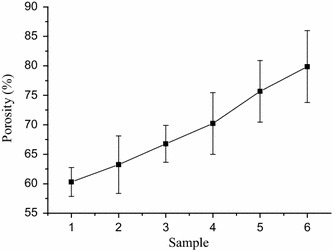
Porosity of micro-HA/CS composite scaffolds. V_composition_ : V_microspheres_ ➀ 50:20 ➁ 50:30 ➂ 50:40 ➃ 50:50 ➄ 50:60 ➅ 50:70

Porosity of micro-HA/CS composite scaffolds is showed in Fig. 7. Porosity measurement studies of composite scaffolds indicated a high porosity with porogen increased. The addition of porogen increased the porosity of scaffolds, this perhaps because increased porogen formed interconnected pore and decreased CS volume.

### Water absorption rate

**Fig. 8:**
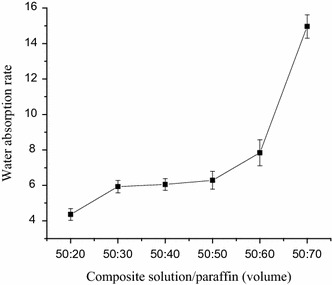
Water absorption of composite scaffolds

The water absorption rate of micro-HA/CS composite scaffolds is showed in Fig. 8. The addition of porogen increased the water absorption of scaffolds.

### Modulus analysis

**Fig. 9:**
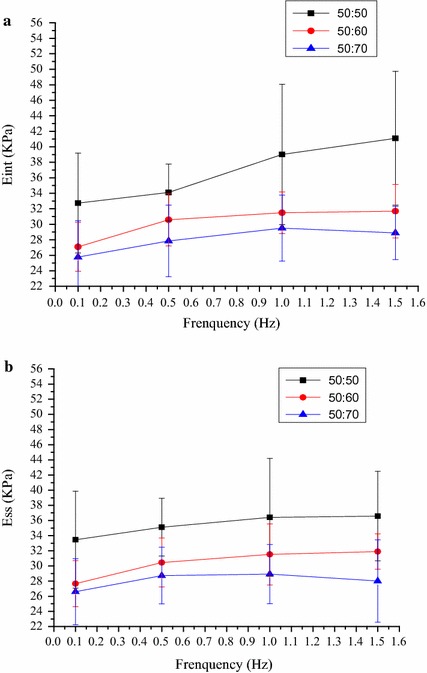
Modulus values for the composite scaffolds. Instantaneous and steady-state modulus values are shown in (**a**, **b**), respectively

Values are reported for testing under compressive strains of 10 % and frequencies of 0.1, 0.5, 1 and 1.5 Hz. The relative stiffness of each sample was measured during both the initial and the sixth hysteresis loops and these data correspond to the modulus values shown in Fig. 9. At each frequency, modulus value is decreased with the paraffin microspheres content, and the 50:50 samples had the largest modulus in the three groups. Statistical analysis revealed that instantaneous modulus values (E_int_) and steady-state modulus values (E_SS_) have no frequency dependence for all the samples, and the modulus values for the samples have no significantly different among three groups (P > 0.05).

### Hysteresis

**Fig. 10:**
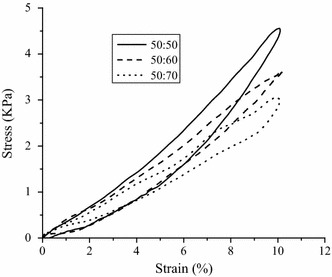
Hysteresis loops during the sixth loading cycle in 1 Hz, 10 % sinusoidal strain for samples

**Fig. 11:**
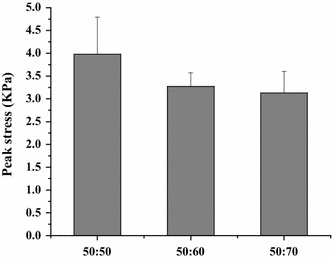
Maximum stress values for each group during the sixth compression cycle. Show no significance difference in the peak stress of the three samples

**Fig. 12:**
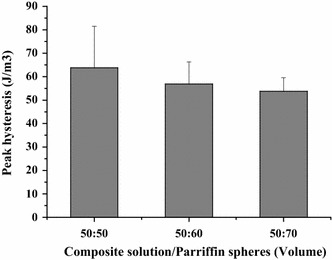
The hysteresis values during the first compression cycle of sample. Show no significance difference in the peak hysteresis of the three samples

**Fig. 13:**
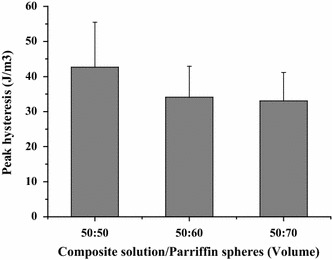
The hysteresis values during the sixth compression cycle of sample. Show no significance difference in the peak hysteresis of the three samples

**Fig. 14:**
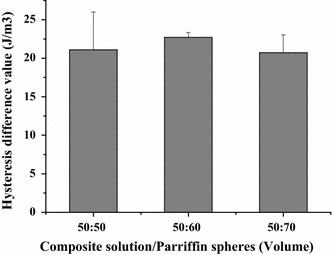
Hysteresis analysis. Hysteresis difference values between the sixth compression cycle and the initial cycle for three samples. Results are not significantly different between three samples

All the samples tested displayed clear hysteresis loops, although the size and shape of the loops varied with porogen content. Representative hysteresis loops corresponding to the first loading cycle are shown in Fig. 10. Comparative peak stresses during the sixth compression cycle are shown in Fig. 11, and peak hysteresis values during the first and the sixth compression cycle are shown in Figs. 12 and 13, and show no significance difference in the peak stresses and peak hysteresis of the three samples. Figure 14 shows that the hysteresis difference values between the sixth compression cycle and the initial cycle for three samples was similar, with no statistically significant differences.

## Discussion

The scaffold provides the general shape and structure of the tissue to be replaced and must promote cell adhesion and subsequent tissue growth by allowing the diffusion of nutrients and cells throughout the scaffold [24]. The interconnected pore is very important in controlling cell seeding and distribution in the scaffolds.The scaffolds structure, pore size and morphology can be easily adjusted by porogens. This study was carried out to fabricate scaffolds with well-controlled pore connectivity by paraffin microspheres as porogen, and to evaluate the scaffolds’ properties.

TCB powder was fabricated from porcine cancellous bone by degreasing, deproteining. The FTIR analysis in combination with XRD analysis clearly indicated the TCB powder was pure hydroxyapatite phase and no other phases of calcium phosphate were formed. To obtain HA particles with uniform particle size, the TBC was being ball milled. The results showed that the particle size distribution of HA particles is relatively uniform, which shows that the appropriate milling parameters was important to the distribution of HA particles. Stirring speed could control the size of paraffin microspheres, which determines the pore size of the scaffold. The obtained paraffin microspheres with diameter of 160 ~ 330 µm were suitable for the preparation of bone scaffolds.

The SEM results showed that the pore number of the scaffolds increased and the pores became interconnected with the increase of porogen. There were some pores deformed, perhaps the stirring deformed the paraffin during the mixing of solution and porogen.

Water absorption rate of scaffolds is very important, and high water absorption rate will be beneficial to the supply of nutrients material after implantation of the scaffold. The addition of porogen increased the water absorption of scaffolds, this perhaps because increased porogen formed interconnected pore.

In vivo, the trabecular bone is subjected to several loading modalities, but under healthy conditions the most relevant is that of dynamic or cyclic compressive loading [25]. Therefore, testing of the scaffolds was carried out based on this loading modality. The sample was immersed in PBS solution to equilibrate, since the native trabecular bone is filled with fluid [26]. This is also in accordance with other studies on bone scaffolds mechanics which have utilized PBS testing environments [27, 28]. The results revealed that instantaneous modulus values (E_int_) and steady-state modulus values (E_SS_) have no frequency dependence for all the samples, and the modulus values for the samples have no significantly different among three groups (P > 0.05). The porous composite scaffolds exhibited a viscosity-elastic behaviour with good repeatability. It is benefit to study the influence of the mechanical load on the cell of the scaffold.

## Conclusions

CS/HA composite scaffolds with interconnected spherical macropores were fabricated using spherical paraffin as porogen. Dynamic compression tests of CS/HA scaffolds have been evaluated in order to study their potential application as scaffolds for bone tissue regeneration. The porous composite scaffolds exhibited a viscosity-elastic behaviour with a maximum stress of 3–4 kPa. Effect of porogen content on the mechanical behaviour was described for three composite scaffolds. With the increase of porogen, the pore of the scaffolds increased and became interconnected. Cyclic loading of three scaffolds were tested with 10 % strain under four levels of loading frequency, 0.1, 0.5, 1 and 1.5 Hz. At each frequency, modulus value is decreased with the paraffin microspheres content, and the 50:50 samples had the largest modulus in the three groups, but there was no significance difference in the peak stress of the three samples. All the samples tested displayed clear hysteresis loops. There was no significance difference in the peak hysteresis of the three samples, and the hysteresis difference values between the sixth compression cycle and the initial cycle for three samples was similar, with no statistically significant differences. It is benefit to study the influence of the mechanical load on the cell of the scaffold.
